# Continuous Erector Spinae Plane Block for Analgesia after Thoracotomy for Lung Transplantation in an Anticoagulated Patient

**DOI:** 10.1155/2021/6664712

**Published:** 2021-02-26

**Authors:** Mark Mudarth, Veena Satyapriya, John Coffman, Peter DeSocio, Alec Lawrence, Shana Schwartz, Michael Kushelev

**Affiliations:** Department of Anesthesiology, The Ohio State University Wexner Medical Center, Columbus, OH, USA

## Abstract

Lung transplant recipients are at particular high risk for postoperative respiratory failure as a result of poorly controlled pain, inadequate graft expansion, decreased cough, and reliance on systemic opioid therapy. Thoracic epidural and paravertebral blocks have been employed with the goal of improving postoperative pain control, improving pulmonary mechanics, and limiting the need for narcotic administration. These approaches require a needle position in proximity to the neuraxis and may cause significant hypotension that is poorly tolerated in transplant patients. Additionally, the use of anticoagulation or underlying clotting disorder limits the use of these regional blocks because of the concern of hematoma and subsequent neurologic injury. Ultrasound-guided continuous erector spinae plane (ESP) block has been shown to be efficacious for pain control following thoracotomy but has had minimal investigations following lung transplantation. In this study, we describe the effective use of a continuous erector spinae plane block to provide analgesia in a postoperative lung transplant recipient receiving systemic anticoagulation. The use of an ESP block with a more superficial needle tract that is further removed from the neuraxis allowed for a greater safety profile while providing efficacious pain control, decreased reliance on systemic narcotics, and improved oxygen saturation. The ESP block was effective in this case and thus may be a valuable alternative following lung transplantation for patients who are not candidates for thoracic epidural or paravertebral approaches.

## 1. Introduction

Thoracotomy for lung transplantation is associated with poorly controlled postoperative pain for a variety of reasons including preoperative deconditioning, elevated anxiety levels, established tolerance to analgesic medications, and dosing limitations of opioids as to avoid hemodynamic compromise [1]. While thoracic epidural analgesia and thoracic paravertebral blocks are commonly considered first-line options, there are instances in which they may be relatively contraindicated. In the setting of systemic anticoagulation, a continuous erector spinae plane (ESP) block has been shown to be a safe and effective alternative in providing analgesia following thoracotomy [2, 3]. For lung transplantation recipients, there is an increasing emphasis on early mobility, progression to independent function, and aggressive oxygen titration in the immediate posttransplantation period [4]. Pain control can play an important role in pulmonary function following thoracotomy because inadequate analgesia prevents deep breathing and graft expansion, which coupled with poor cough and reduced mucociliary clearance, and can result in atelectasis, hypoxemia, pneumonia, graft failure, and prolonged mechanical ventilation [5, 6].

## 2. Case Report

**Figure 1:**
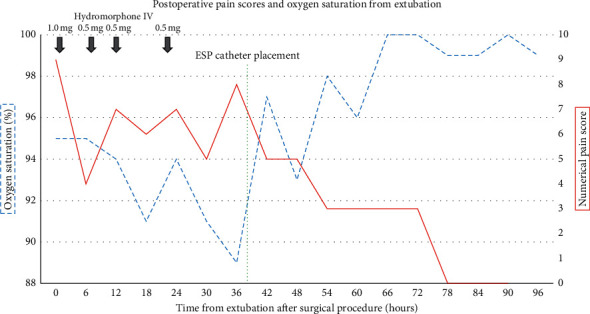
Postoperative pain scores, oxygen saturation, and intravenous hydromorphone consumption from extubation through recovery. ESP, erector spinae plane; IV, intravenous.

We describe the successful use of a continuous ESP block for a 57-year-old woman who underwent left pneumonectomy and single lung transplantation via posterolateral thoracotomy incision complicated by atrial fibrillation requiring cardioversion and subsequent therapeutic anticoagulation with heparin. The patient did not receive any preoperative regional techniques as per our typical institutional practices for lung transplantation at the time. The patient developed atrial fibrillation during surgery that was not responsive to repeated attempts at electrical cardioversion and was started on an amiodarone infusion at 1 mg/min immediately postoperatively. Atrial fibrillation persisted, and 8 hours postoperatively, a heparin infusion was initiated at 15 units/kg/hr in anticipation of repeated cardioversion with a goal of activated partial thromboplastin time of 2 times greater than baseline. Three hours after surgery, the patient was extubated and transitioned to heated Hi-Flow Nasal Cannula (HFNC) at fraction of inspired oxygen (FiO_2_) of 65% at 50 liters (L) to maintain her oxygen saturation >88% on continuous pulse oximetry. For the first 24 hours postextubation, the patient maintained adequate oxygen saturation on HFNC with a range of 40–70 L and FiO2 of 45–65% without attempting to ambulate. Shortly following extubation, the patient reported uncontrolled pain, despite repeated administration of intravenous opioids (pain scores 5/10 at rest and 10/10 with exertion), with resultant difficulty participating in adequate pulmonary hygiene (Figure 1). Pain scores were assessed as per standard nursing protocol every 4 hours on a 0–10 numerical rating scale, and oxygenation was assessed with continuous pulse oximetry.

Following extubation, the patient was continued on her baseline dose of 10 mg oxycodone every 8 hours for chronic back pain and was additionally administered intravenous Toradol 15 mg every 6 hours and oral acetaminophen 650 mg every 6 hours for the first 7 postoperative days. Thirty-six hours following extubation, the patient continued to exhibit difficulty to control pain (10/10 with ambulation) and continued to have respiratory compromise despite HFNC of 60–70% at high flows. During a 130-foot walk, the patient was unable to maintain oxygenation saturation levels above 87% despite continued use of HFNC with FiO_2_ of 70% and 70 L. In the first 36 hours postoperatively, the patient consistently reported pain scores ranging from 8 to 10. After obtaining informed consent, an ESP catheter was performed as described by Forero et al. [7]. At the time of ESP catheter placement, systemic anticoagulation with heparin was maintained with a therapeutic partial thromboplastin time (PTT) of 79.7 seconds. A high frequency linear ultrasound transducer (BK Medical Flex Focus 400, Peabody, MA) was positioned in a longitudinal parasagittal orientation 3 cm lateral to the T5 spinous process, and the erector spinae muscle was identified. A 17 gauge, 5 cm Tuohy needle (Arrow® StimuCath® Teleflex Medical, Morrisville, NC) was advanced from cephalad to caudad, in-plane to the ultrasound probe, in order to achieve a final position deep to the erector spinae muscle. This location was confirmed with visualization of 0.2% ropivacaine 15 ml spreading cranially and caudally beneath the erector spinae muscle. Subsequently, a 19 gauge, single orifice, wirewound catheter (Arrow) was advanced, such that 3 cm remained beneath the erector spinae muscle. Following a negative test dose, an additional 0.2% ropivacaine 10 ml was administered through the catheter. A continuous ESP infusion utilizing an elastomeric pump was then initiated: 0.2% ropivacaine at 8 ml/hour continuous infusion; the patient controlled a bolus dose of 0.2% ropivacaine at 5 ml every 30 minutes and a 16 ml/hour lockout dose. The ESP catheter and infusion were maintained for 92 hours, during which time, the patient endorsed lower pain scores, no additional intravenous opioid medications for breakthrough pain, improved oxygenation, and stronger cough (Figure 1). The patient continued to demonstrate paroxysmal atrial fibrillation and was thus continued on heparin infusion with a therapeutic range of partial thromboplastin time for the first 6 postoperative days and was then transitioned to warfarin therapy. Heparin infusion was not interrupted for catheter removal. No apparent complications related to the ESP catheter were noted following catheter removal on physical examination during the remainder of the patients' lengthy hospital stay.

## 3. Discussion

Thoracic epidural analgesia and thoracic paravertebral block are mainstays for management of postthoracotomy pain, but the ESP block can serve as an effective alternative [8–11]. A growing adoption of the fascial plane block has led to a growing body of literature that has demonstrated efficacy of ESP blocks. The ESP block has been used successfully for various abdominal and thoracic surgical procedures including hepatectomy, bariatric surgery, mastectomy, and cardiac surgery as well as chronic thoracic pain conditions [12–16]. For major thoracic surgical procedures, the ESP block appears to offer visceral, in addition to somatic analgesia by allowing for anterior spread of local anesthetic into the paravertebral and epidural space thus blocking ventral and dorsal rami of the thoracic spinal nerves [13, 17]. An ESP block at the T5 transverse process in cadavers has been shown to allow for significant spread of local anesthetic in the craniocaudal plane between T3 and L2 [18]. Especially pertinent for a lung transplantation patient, ESP has been shown to improve inspiratory capacity in trauma patients with rib fractures [19].

An ESP block requires a relatively superficial needle path targeting a myofascial plane deep to the erector spinae muscle allowing for easier sonographic identification of the pertinent anatomy. Performing an ESP block may present a greater safety profile by virtue of being further removed from the pleura and spinal cord with less-associated hemodynamic disturbance and potentially less risk of bleeding leading to neurologic compromise. While there continues to be a lack of convincing data regarding the risk of hematoma formation for deeper blocks with a wide range of anticoagulation medications, the consequences of hematoma leading to cord compression are less significant as one travels further from the neuraxis. Whether to consider the ESP as a superficial or deep block in regards to risk of hematoma formation is unknown secondary to a lack or randomized controlled trials. Of note, one small case series reported the use of ESP in 5 patients with an international normalized ratio >1.5 without any recognizable bleeding or neurologic complications [20].

While the available literature for the use ESP for patients undergoing thoracic surgical procedures is still limited, a growing body of evidence suggests that ESP is a viable alternative for thoracic surgical procedures. Further studies comparing ESP catheters to thoracic epidural and paravertebral approaches are needed to assess the optimal analgesic technique for patients undergoing lung transplantation.

## 4. Conclusion

In this case, the use of a continuous erector spinae block improved pain control following single lung transplantation as evidenced by decreased pain scores and opioid administration while concomitantly improving oxygenation. Immediate recovery following lung transplantation is facilitated by adequate pain control thus allowing for improved respiratory mechanics. Considering that thoracic epidural and paravertebral blocks are frequently not a viable option in this patient population, we propose that an ESP block can serve as an effective regional technique that minimizes systemic opioids while improving deep breathing and graft expansion.

## Data Availability

The data used to support the findings of this study are included within the article.

## References

[1] Feltracco P., Barbieri S., Milevoj M. (2010). Thoracic epidural analgesia in lung transplantation. *Transplantation Proceedings*.

[2] Adhikary S. D., Prasad A., Soleimani B., Chin K. J. (2019). Continuous erector spinae plane block as an effective analgesic option in anticoagulated patients after left ventricular assist device implantation: a case series. *Journal of Cardiothoracic and Vascular Anesthesia*.

[3] Fang B., Wang Z., Huang X. (2019). Ultrasound-guided preoperative single-dose erector spinae plane block provides comparable analgesia to thoracic paravertebral block following thoracotomy: a single center randomized controlled double-blind study. *Annals of Translational Medicine*.

[4] Wickerson L., Rozenberg D., Janaudis-Ferreira T. (2016). Physical rehabilitation for lung transplant candidates and recipients: an evidence-informed clinical approach. *World Journal of Transplantation*.

[5] Richardson J., Sabanathan S., Shah R. (1999). Post-thoracotomy spirometric lung function: the effect of analgesia. a review. *Journal of Cardiovascular Surgery (Torino)*.

[6] Sabanathan S., Eng J., Mearns A. J. (1990). Alterations in respiratory mechanics following thoracotomy. *Journal of the Royal College of Surgeons of Edinburgh*.

[7] Forero M., Adhikary S. D., Lopez H., Tsui C., Chin K. J. (2016). The erector spinae plane block. *Regional Anesthesia and Pain Medicine*.

[8] Forero M., Rajarathinam M., Adhikary S., Chin K. J. (2017). Continuous erector spinae plane block for rescue analgesia in thoracotomy after epidural failure. *A & A Case Reports*.

[9] Wilson J. M., Lohser J., Klaibert B. (2018). Erector spinae plane block for postoperative rescue analgesia in thoracoscopic surgery. *Journal of Cardiothoracic and Vascular Anesthesia*.

[10] Kelava M., Anthony D., Elsharkawy H. (2018). Continuous erector spinae block for postoperative analgesia after thoracotomy in a lung transplant recipient. *Journal of Cardiothoracic and Vascular Anesthesia*.

[11] Joshi G. P., Bonnet F., Shah R. (2008). A systematic review of randomized trials evaluating regional techniques for postthoracotomy analgesia. *Anesthesia & Analgesia*.

[12] Ahıskalıoğlu A., Alıcı H. A., Çiftçi B., Celik M., Karaca Ö. (2019). Continuous ultrasound guided erector spinae plane block for the management of chronic pain. *Anaesthesia, Critical Care and Pain Medicine*.

[13] Chin K. J., Malhas L., Perlas A. (2017). The erector spinae plane block provides visceral abdominal analgesia in bariatric surgery. *Regional Anesthesia and Pain Medicine*.

[14] Kang R., Chin K. J., Gwak M. S. (2019). Bilateral single-injection erector spinae plane block versus intrathecal morphine for postoperative analgesia in living donor laparoscopic hepatectomy: a randomized non-inferiority trial. *Regional Anesthesia & Pain Medicine*.

[15] Veiga M., Costa D., Brazão I. (2018). Erector spinae plane block for radical mastectomy: a new indication?. *Revista Española de Anestesiología y Reanimación (English Edition)*.

[16] Krishna S. N., Chauhan S., Bhoi D. (2019). Bilateral erector spinae plane block for acute post-surgical pain in adult cardiac surgical patients: a randomized controlled trial. *Journal of Cardiothoracic and Vascular Anesthesia*.

[17] Schwartzmann A., Peng P., Maciel M. A., Forero M. (2018). Mechanism of the erector spinae plane block: insights from a magnetic resonance imaging study. *Canadian Journal of Anesthesia/Journal Canadien D’anesthésie*.

[18] Adhikary S. D., Pruett A., Forero M., Thiruvenkatarajan V. (2018). Erector spinae plane block as an alternative to epidural analgesia for post-operative analgesia following video-assisted thoracoscopic surgery: a case study and a literature review on the spread of local anaesthetic in the erector spinae plane. *Indian Journal of Anaesthesia*.

[19] Adhikary S. D., Liu W. M., Fuller E., Cruz‐Eng H., Chin K. J. (2019). The effect of erector spinae plane block on respiratory and analgesic outcomes in multiple rib fractures: a retrospective cohort study. *Anaesthesia*.

[20] Galacho J., Veiga M., Ormonde L. (2020). Erector spinae plane block and altered hemostasis: is it a safe option? -a case series-. *Korean Journal of Anesthesiology*.

